# Implementation and Outcomes of a Comprehensive Tobacco Free Workplace Program in Opioid Treatment Centers

**DOI:** 10.3390/ijerph19010239

**Published:** 2021-12-26

**Authors:** Matthew Taing, Vijay Nitturi, Tzuan A. Chen, Bryce Kyburz, Isabel Martinez Leal, Virmarie Correa-Fernández, Ezemenari M. Obasi, Teresa Williams, Kathleen Casey, Daniel P. O’Connor, Litty Koshy, Maggie Britton, Kelli Drenner, Lorraine R. Reitzel

**Affiliations:** 1 Department of Psychological, Health & Learning Sciences, The University of Houston, 3657 Cullen Blvd Stephen Power Farish Hall, Houston, TX 77204, USA; mtaing@central.uh.edu (M.T.); vnitturi@central.uh.edu (V.N.); tchen3@central.uh.edu (T.A.C.); imarti31@central.uh.edu (I.M.L.); vcorreaf@central.uh.edu (V.C.-F.); emobasi@central.uh.edu (E.M.O.); mkbritto@central.uh.edu (M.B.); kldrenne@central.uh.edu (K.D.); 2 Health Research Institute, The University of Houston, 4349 Martin Luther King Blvd., Houston, TX 77204, USA; dpoconno@central.uh.edu (D.P.O.); littyk92@gmail.com (L.K.); 3 Integral Care, 1430 Collier Street, Austin, TX 78704, USA; bryce.kyburz@integralcare.org (B.K.); teresa.williams@integralcare.org (T.W.); kathleen.casey@integralcare.org (K.C.); 4 Department of Health & Human Performance, The University of Houston, 3875 Holman Street, Garrison Gymnasium, Room 104, Houston, TX 77204, USA

**Keywords:** tobacco control, opioid addiction, substance use, implementation science

## Abstract

Tobacco use is exceedingly high among individuals receiving care for opioid addiction, but not commonly addressed by clinicians in treatment settings. Taking Texas Tobacco Free (TTTF) is a comprehensive tobacco-free workplace (TFW) program that builds treatment centers’ capacity to address tobacco use with evidence-based tobacco cessation policies and practices. Here, we examine the process and outcomes of TTTF’s implementation within 7 opioid addiction centers. Program goals were structured according to the RE-AIM framework. Pre- and post-implementation data were collected from client facing and non-client facing employees to assess changes in education, training receipt, knowledge, and intervention behaviors, relative to program goals. Centers reported tobacco screenings conducted and nicotine replacement therapy (NRT) delivered through 6 months post-implementation. Overall, 64.56% of employees participated in TTTF-delivered tobacco education, with a 54.9% gain in tobacco control and treatment knowledge (*p* < 0.0001), and significant increases in exposure to education about tobacco use and harms among individuals with opioid use disorder (*p* = 0.0401). There were significant gains in clinicians’ receipt of training in 9/9 tobacco education areas (*ps* ≤ 0.0118). From pre- to post-implementation, there were mean increases in the use of the 5A’s (ask, advise, assess, assist, and arrange) and other evidence-based interventions for tobacco cessation, with statistically significant gains seen in NRT provision/referral (*p* < 0.0001). Several program goals were achieved or exceeded; however, 100% center participation in specialized clinical trainings was among notable exceptions. One program withdrew due to competing pandemic concerns; all others implemented comprehensive TFW policies. Overall, TTTF may have improved participating opioid treatment centers’ capacity to address tobacco use, although study limitations, including lower post-implementation evaluation response rates, suggest that results require replication in other opioid addiction treatment settings.

## 1. Introduction

The opioid epidemic has garnered national attention in recent years. However, its toll is modest compared to tobacco; for example, in 2018, there were an estimated 46,802 opioid-related deaths, as opposed to the 480,000 tobacco-related deaths occurring annually in the United States (U.S.) [1,2]. While both epidemics present unique challenges to public health, tobacco control efforts may be overshadowed by steadily declining smoking rates among the general population [3,4]. Despite this decline, smoking continues to disproportionately affect marginalized subpopulations, especially those with substance use disorders [5]. In particular, individuals with opioid use disorders (OUDs) have disproportionately higher smoking rates compared to the general population; data suggest the prevalence of smoking among this demographic is nearly 6-fold that of the general U.S. adult population (~84–94% vs. 14%, respectively) [5,6]. This disparity among individuals with OUDs compounds the burden of smoking-related morbidity and mortality and increases risks for opioid relapse and poorer quality of life [7,8]. 

The lack of tobacco control efforts among smokers with OUD may reflect multi-faceted challenges to smoking cessation [7]. For example, smokers with OUD often have limited access to evidence-based smoking cessation treatment and face increased exposure to treatment settings with pro-smoking social norms [7]. Likewise, many OUD treatment programs fail to offer sustained, evidence-based treatments for tobacco use, such as counseling and/or pharmacotherapy [7]. Indeed, numerous national surveys found that <40% of opioid programs provided smoking cessation counseling and less than one-third provided pharmacotherapy [9,10,11,12,13,14]. Smokers with OUDs also face individual-level challenges such as a greater risk perception of other medical illnesses over smoking-related health concerns, psychiatric comorbidities, and increased pain intensity during nicotine withdrawal [7,15,16,17,18,19,20]. Moreover, concurrent use of opioids and nicotine products can present treatment problems due to interactions between opioid and nicotine-cholinergic neurotransmitter systems [21]. Given these unique challenges, efforts are needed to effectively address concurrent tobacco use among individuals with OUDs. 

The implementation of a comprehensive tobacco-free workplace program in OUD treatment settings is under-studied; such programs simultaneously address multiple-levels of influence on tobacco use with evidence-based tobacco control policies and practices [22]. The inclusion of organizational-level policy changes to foster tobacco-free workplace environments and stakeholder education on the harms of tobacco use may potentially lessen the effects of social smoking cues and decrease pro-smoking social norms within these settings [23]. This, in addition to building capacity for individual-level clinical treatment of tobacco use through clinician training and resource provision, may improve the standard of care provided and lead to longer-term gains in tobacco control among relevant stakeholders in OUD treatment settings.

Taking Texas Tobacco Free (TTTF) is a comprehensive, evidence-based tobacco-free workplace program seeking to increase the capacity of partnering agencies to address tobacco use. TTTF’s multicomponent approach to implementation includes: (1) providing education for all employees regarding the risks of tobacco use and benefits of quitting, (2) training clinicians on screening and treating tobacco dependence; (3) implementing an enforceable tobacco-free workplace policy; and (4) providing nicotine replacement therapy (NRT) and other resources (e.g., permanent workplace signage and passive dissemination materials) to facilitate tobacco cessation [24,25,26]. From 2013–2018, TTTF was successfully implemented in 23 local mental health authorities—centralized administrative state agencies that oversee hundreds of individual mental health treatment centers across the state of Texas [24,27,28,29,30]. Pre- to post-implementation results demonstrated gains in both clinical and non-clinical employee education, increased use of tobacco use assessments and evidence-based cessation practices, increased rate of tobacco cessation among non-clinical employees, and higher demand for NRT [28]. In 2017, TTTF was scaled-out to smaller, more independent, less well-resourced, and more population- or disorder-specific, non-profit substance use treatment centers [25,31] and community agencies [32]. This effort included implementation within several OUD treatment centers, which are the subject of this report. 

There is limited to no literature to inform effective implementation of a tobacco-free workplace program in OUD treatment settings. Despite positive outcomes from prior TTTF implementations [26,28], the collection of direct empirical evidence regarding implementation outcomes relative to stated goals is necessary when expanding to new treatment settings [33]. As such, the purpose of this study is to examine processes and outcomes of the TTTF program’s implementation within standalone OUD treatment settings. 

## 2. Materials and Methods

### 2.1. Participants

#### 2.1.1. Agency Recruitment

Our funding permitted program implementation in >18 enrolled Texas substance use treatment centers. Any substance use treatment centers that was willing to fully participate in the program was eligible for inclusion; recruitment was not restricted to opioid treatment programs. Recruitment was accomplished through multiple means, including via professional organization listservs, postal mail solicitation, email solicitation by the team and prior TTTF partners, word of mouth, our website, etc. Centers were accepted into the program on a rolling, first-come, first-served basis between 2017 and 2020.

#### 2.1.2. Participating Agencies

Overall, we enrolled 19 substance use treatment centers into the program; 7 of these were stand-alone OUD treatment centers. These OUD treatment centers spanned the Houston (*n* = 3) and Dallas (*n* = 4) metropolitan areas and served clients across 5 counties in Texas (Harris, Dallas, Tarrant, Grayson, Nueces) and are the subject of this report. Of Texas’s 254 counties, Harris, Dallas, and Tarrant are among the largest, with approximately 9.5 million residents between them; Nueces is the 16th largest with a population of 362,294; and Grayson is the 34th largest with 136,212 residents [34]. 

#### 2.1.3. Participating Clinicians and Their Reach

Participating OUD treatment centers reported 71 client-facing staff and 8 non-client facing staff. These groups, considered jointly, are hereafter referred to as “employees.” Client-facing staff (i.e., clinicians) roles and titles varied, as some provided direct services (e.g., prescribing tobacco treatment medications) whereas others provided case management or counseling. Non-client facing staff included leadership, human resources professionals, etc. The participating treatment centers had an estimated 116,087 annual client contacts (Table 1).

### 2.2. Procedures

Study procedures were approved by the University of Houston’s IRB. Enrollment began when leadership signed memorandums of understanding, which detailed program requirements and participant responsibilities. Within ~2–3 weeks following enrollment, pre-implementation data were gathered online from leadership and employees, including information on existing tobacco-related policies, number and type of employees at these centers, their smoking status, receipt of tobacco control/treatment education, and clinician behaviors in addressing tobacco use with clients. 

The next steps entailed the provision of 1.5-to-2 h training sessions on tobacco control and treatment, tailored for tobacco use among individuals with OUD, to all employees. While these trainings were highly recommended to all employees, they were not mandated in consideration of feasibility and logistics issues inherent to these frequently understaffed and under-resourced centers [35,36]. These trainings were conducted on site by TTTF staff pre-COVID (Centers 1–5) and virtually post-COVID (Centers 6–7). In both cases, pre- and post-training tests were administered to attendees to assess knowledge gain. Clinicians were additionally provided specialized training, which consisted of: (1) sponsorship for 1–2 clinicians per center to attend a 5-day Certified Tobacco Treatment Specialist training [37], and (2) provision of a 7.5 h Motivational Interviewing training by TTTF staff to help clinicians better understand how to motivate their clients to begin and/or sustain tobacco quit attempts. Each center also received passive dissemination materials (e.g., posters and rack cards) to display in their facilities and distribute to clients with messages encouraging clients to ask their treatment providers for more information on how to quit smoking, along with the Tobacco Quitline phone number and online resources. TTTF also provided all partnering centers with a NRT starter kit (typically valued at ~$3200 to ~$16,000 per center, based on clientele/employee volume) for distribution to clients and employees. It is notable that none of the completing centers dispensed NRT prior to TTTF program implementation. Finally, tobacco-free workplace signage was provided by TTTF to place around campuses to signify the official transition to a tobacco-free workplace or modification of an existing partial policy. TTTF’s requirement for comprehensive policies was that all tobacco use (including e-cigarettes) should be prohibited both inside buildings and on all center property. 

Following TTTF implementation, online post-implementation surveys were distributed to employees and clinicians-only to measure the effects that trainings and other TTTF procedures had on center employees and their work with clients. Active program implementation within participating centers, from enrollment through post-implementation data collection, was ~7.3 months long on average (range = 6–8 months). Data on conducted tobacco use assessments and distributed NRT were collected quarterly through 6 months post-implementation to measure sustainment. 

### 2.3. Program Goals

Programs goals were aligned to the Reach, Effectiveness, Adoption, Implementation, and Maintenance (RE-AIM) framework, which provides a useful model for translating research into practice [38]. Thresholds for study measures to evaluate implementation success were developed based on our previous implementation in local mental health authorities [24,26].

Reach (Goal 1) was conceptualized as: (1) educating >60% of employees at each center with our 1.5–2 h trainings; (2) achieving statistically significant increases in employees’ exposure to educational concepts from pre- to post-implementation; and embedding specialized knowledge into each center by: (3a) having at least 1 clinician attend the Certified tobacco Treatment Specialist training, and (3b) by having at least 60% of clinicians attend the Motivational Interviewing training. 

Effectiveness (Goal 2) was conceptualized as: (1) achieving a 50% employee knowledge gain from the provided TTTF education; and (2) a > 2% reduction in employee tobacco use. This project was focused on clinician and employee behavior change and did not include direct data collection with clients about their tobacco quit attempts or abstinence outcomes. This was because clinicians’ routine provision of empirically supported interventions for tobacco cessation are known to subsequently lead to increased client quit rate [39]. Thus, effectiveness as related to clientele was primarily measured by the reach of our evidenced-based education and the adoption, implementation, and maintenance of evidence-based practices for tobacco use disorders. This approach—measuring impact of known-efficacious interventions by outcomes other than quit rates—has been successfully used with other large scale tobacco control interventions—an acceptable practice as evidenced by publication in high impact journals [40,41]. 

Adoption (Goal 3) was envisioned as: (1) >80% of enrolled centers completing the TTTF program; (2) 100% of program completers having implemented an enforceable tobacco-free workplace policy with prominent signage that disallowed tobacco use anywhere on the workplace, including on its grounds. 

Implementation (Goal 4) was envisioned as achieving >65% of responding clinicians reporting the regular provision of tobacco use screening and empirically based interventions to clients by post-implementation. This included: (1) the 5A’s of tobacco screening (Asking about tobacco use) and intervention (Advising patients to quit; Assessing willingness to quit; Assisting them to quit; Arranging follow-up); and (2) behavioral counseling, NRT or referral for NRT, and non-nicotine-based medications (e.g., Chantix) or referral for medication.

Maintenance (Goal 5) was conceptualized as >80% of completing centers being compliant with: (1) the provision of reports of tobacco use assessments administered, and (2) NRT dispensed within the 6 months following program implementation. 

### 2.4. Measures

#### 2.4.1. Tobacco Control and Treatment Education Reach (Goal 1, Reach)

Tobacco education reach was collected with pre- and post-implementation surveys administered to all employees and to clinicians-only, respectively. These surveys were distributed within each center by a center-designated program champion. Survey items were face-valid, investigator-generated queries. The employee survey items relevant to this goal consisted of 2 items: “In the last 12 months, have you received any education regarding the hazards of smoking?” and “In the last 12 months, have you received any training regarding the hazards of smoking and benefits of quitting that are specific to individuals with substance use disorders?” (options = yes or no). The clinician survey consisted of 9 additional items that queried receipt of specialized training and use of evidence-based smoking cessation interventions. Data were collected anonymously to promote honesty and to minimize any perception of risk to employment status based on responses. 

Program records were used to evaluate the number of clinicians who attended Certified Tobacco Treatment Specialist and Motivational Interviewing trainings. 

#### 2.4.2. Tobacco Control and Treatment Education Knowledge Gain and Employee Tobacco Use (Goal 2, Effectiveness)

A 10-item test was administered before and after each education session to measure knowledge gained from the training. Items included: “Which of these tobacco treatment medications requires a prescription?” (response options = nicotine patch, inhaler, lozenge, gum, or “all of the above”), and “Smoking cessation interventions were associated with ____ increased likelihood of long-term alcohol and drug abstinence following substance abuse treatment” (response options = 15%, 20%, 25%, and 30%). The test items directly reflected content of the educational session. Data were collected anonymously to promote honesty and to minimize any perception of risk to employment status based on responses. 

Employee tobacco use was measured on pre-implementation and post-implementation employee surveys with the following items: “Currently, how often do you smoke cigarettes?” (options = I do not smoke or have quit completely, some days, every day, weekends, or only when I drink alcohol) and “Do you regularly use any of the following products?” (options = snus; roll your own cigarettes; tobacco from a hookah or waterpipe; dissolvable products such as Ariva/Stonewall/Camel/Camel Orbs/Camel sticks; electronic cigarettes or E-cigarettes such as Fin, NJOY, Blu, e-Go, and Vuse; cigars, little cigars/cigarillos/bidis; chewing tobacco/dip/snuff; and I do not use any other tobacco products). Responses to these 2 items were collated to determine employee tobacco use status at pre- and at post-implementation (options = tobacco user vs. not a tobacco user). 

#### 2.4.3. Program Completion and Tobacco-Free Workplace Policy Implementation (Goal 3, Adoption) 

Program completion and tobacco-free workplace policy implementation was assessed using program records. Specifically, program completion was defined as accepting the following: education for staff, training for clinicians, NRT, workplace signage (if permissible based on center location) and passive dissemination materials (e.g., posters, rack cards, etc.). The study team kept a shared spreadsheet to indicate when these education and training sessions took place, as well as the NRT, signage, and passive dissemination materials ordered. The implementation of a tobacco-free workplace policy was overseen by the TTTF project manager, who confirmed an existing policy or assisted with and reviewed development of a new policy. The project manager worked from a program implementation timeline to ensure that if a new policy were developed, it was approved by the board or human resources (if applicable) and communicated to all staff via an email highlighting the center’s participation in the TTTF program. Furthermore, employees were trained during program implementation on how to have conversations with clients/guests/visitors violating the policy to ensure reasonable enforcement.

#### 2.4.4. Clinician Intervention on Tobacco Use and Clients (Goal 4, Implementation) 

Clinician tobacco intervention practices were assessed pre- and post-implementation with clinician surveys. Items assessed included the use of the 5A’s for smoking (Ask, Advise, Assess, Assist, and Arrange) and other empirically supported treatments for tobacco use [39]. Items included “What types of treatment do you typically provide for smokers or other tobacco users?” with options that included behavioral counseling, NRT or referral for NRT, and non-nicotine-based medications (e.g., Chantix) or referral for medication. Data were collected anonymously for the aforementioned reasons. 

#### 2.4.5. Post-Implementation Tobacco Use Assessment and Nicotine Replacement Therapy (Goal 5, Maintenance) 

TTTF staff collected quarterly data via an online survey regarding the number of tobacco use assessments conducted and number of NRT boxes distributed to clients and employees during the 6 months after the TTTF program was implemented in each center, respectively. 

### 2.5. Analytic Plan

Descriptive statistics regarding outcomes of interest were calculated. Pre- to post-measurements were aggregated by center, not the individual respondent level. Data missingness due to program withdrawal or failure to administer measures was assessed. Handling of this form of missingness in analyses was determined a priori, using a commonsense approach whereby all centers were included in analyses when both pre- and post-data were available. In cases where post-data was missing due to withdrawal or failure to administer measures, only centers providing complete data were included in analyses. In this study, the calculations of the survey response rate and item response rate were defined as follows: survey response rate = number of respondents who started this survey/number of all eligible sample members; item response rate = number of respondents who responded to this item/number of respondents who started this survey.

Tobacco control and treatment education reach, for all employees and clinicians-only, were summarized using descriptive statistics. Achievement of statistical significance was not a part of most program goals (see Section 2.3); however, we assessed significance whenever possible in the interest of thorough reporting. Statistical significance was assessed using Cochran–Mantel–Haenszel tests, based on the need to control for implementation site (Goal 1, Reach). Pre- and post-knowledge tests were scored based on the number of correct items (possible range 0–10). Tobacco control and treatment education knowledge gain was measured by percentage comparisons of correct items from the pre- and post-education knowledge test. Differences of knowledge gain prior to and after the TTTF training session were examined using an analysis of covariance, controlling for center. The change in employee tobacco use rates was calculated as the percent change in tobacco use rates from pre- to post-implementation. Here, statistical significance of changes over time were examined using Fisher’s exact test (Goal 2, Effectiveness), again in the interest of thorough reporting.

The proportion of program completers and centers adopting/refreshing a tobacco-free workplace policy were obtained from program records (Goal 3, Adoption). Changes in clinician intervention on tobacco use with clients were examined with descriptive statistics. Statistical significance of change over time was assessed using Cochran–Mantel–Haenszel tests (Goal 4, Implementation), for thorough reporting. Quarterly report data on tobacco use assessment and NRT administration (Goal 5, Maintenance) were summed, respectively. All statistical analyses were conducted using SAS version 9.4 [42].

## 3. Results

### 3.1. Data Missingness

Of the 7 OUD treatment centers that enrolled in this program, one withdrew from participation due to competing priorities and staff shortages due to the pandemic (Center 7). This withdrawal occurred following the provision of the tobacco control and treatment education/training; thus, employees completed pre-implementation surveys. However, employees (*n* = 2) who attended the education/training delivery did not participate in pre- or post-knowledge tests. An additional center (Center 1) did not provide post-implementation employee data, citing very high rates of employee turnover since the initial assessment that would impact comparisons with pre-implementation data. However, high turnover rates were not seen amongst clinicians; post-implementation clinician data were provided by this center.

### 3.2. Tobacco Contol and Treatment Education Reach (Goal 1, Reach)

The first component of this program goal was to reach >60% of employees at each center with tobacco control and treatment education. Overall, 51 employees (51/79 = 64.56%) participated in these trainings between the 7 treatment centers, indicating achievement of the reach part of the goal.

Secondly, we strove for statistically significant increases in exposure to education concepts among employees. Due to data missingness, analyses of clinician data were conducted with 6 centers whereas analyses of non-client facing staff were conducted with 5 centers. Although increases in exposure to education concepts were realized over time for both clinicians and non-client facing staff, statistically significant changes were only observed amongst clinicians (Table 2). Thus, this aspect of reach was only partially met.

Thirdly, the goal for 100% center participation in specialized training was only partially achieved. TTTF sponsored 4 clinicians from 3 centers (3/7 centers = 42.86%) to attend Certified Tobacco Treatment Specialist training. Unfortunately, remaining centers could not send a clinician to the training due to competing work demands. Two centers sent clinicians (*n* = 6) for Motivational Interviewing training (28.57%). Centers that did not participate indicated their clinicians already had Motivational Interviewing training or indicated that scheduling conflicts and center capacity precluded attendance. Thus, this aspect of our reach goal—sending 1 or more clinicians to Certified Tobacco Treatment Specialist training and 60% to Motivational Interviewing trainings—was not achieved.

### 3.3. Tobacco Control and Treatment Education Knowledge Gain (Goal 2, Effectiveness)

The 2nd program goal was to achieve a 50% knowledge gain from the tobacco control and treatment education sessions, as evaluated by 46 pre- and 39 post-tests from 6 centers that participated in this aspect of data collection. The average pre-test scores were 5.28 (SD = 1.83) and the average post-test scores were 8.18 (SD = 1.19) out of a possible 10. Overall, knowledge gain ranged from 19.4 to 84.2% with a 54.9% knowledge gain in all 6 centers combined (*p* < 0.001). Thus, this aspect of the 2nd program goal was achieved.

Tobacco using employees decreased by >2% from pre- to post-implementation from 5 centers providing data for both time points. Overall, 9 employees (28.13%) reported being a tobacco user at pre-implementation, compared to 3 employees (13.64%) at post-implementation. The percent change in tobacco use amongst employees over time was 51.40%. These changes, however, were not statistically significant (*p* = 0.3202) and low response rates at post-implementation call into question whether the difference in tobacco use prevalence was truly because there were fewer tobacco users at post-implementation, or simply because there were fewer respondents. Thus, the data are not sufficient to determine whether this part of Goal 2 was met. 

### 3.4. Program Completion and Tobacco-Free Workplace Polict Implemtation (Goal 3, Adoption)

Overall, 6/7 (85.71%) enrolled centers completed the TTTF program, meeting this program goal of maintaining the participation of >80% of centers in the program until its end.

At pre-implementation, only a single center (Center 2) had a pre-existing tobacco-free workplace policy that included e-cigarettes and prohibited tobacco use both indoors and on the workplace grounds. By post-implementation, 100% of centers completing the program (*n* = 6) implemented an enforceable and complete tobacco-free workplace policy. Permanent tobacco-free workplace signage was accepted by all the completing centers except one where this was not practicably feasible. Specifically, the single completing center that did not accept signage from the program was located on the 2nd floor of a building that housed multiple businesses; the overall building already had tobacco control signage and tenants were not authorized to install permanent signage on building grounds.

### 3.5. Clinician Intervention on Tobacco Use with Clients (Goal 4, Implementation)

This program goal (set at the time of project application) was to achieve ≥65% of responding clinicians reporting the use of the 5A’s and other empirically supported treatments for tobacco use by post-implementation survey. There were 36 pre- and 28 post-tests from 6 centers with which to evaluate this goal.

Overall, >65% of clinicians reported using each of the 5A’s at post-implementation (Table 3). However, it is worth noting that the goal of ≥65% was already reported at pre-implementation for Ask, Advise, and Assess; only Assist and Arrange were <65%. Changes from pre- to post-implementation were not statistically significant in any category. The >65% goal of clinician use of other empirically supported interventions for tobacco control was achieved for NRT use or referral/recommendation for such but not for behavioral counseling or the use of non-nicotine medications (Table 3). Thus, this goal was only partially met. Changes from pre- to post-implementation were only statistically significant in the case of NRT or referral/recommendation for such.

### 3.6. Post-Implementation Tobacco Use Assessment and Nicotine Replacement Therapy (Goal 5, Maintenance)

The final program goal was to have centers engage in regular tobacco use assessments and NRT delivery through 6 months post-implementation. In total, participating centers delivered 272 tobacco use assessments post-implementation. Overall, 11 shipments of NRT (1872 boxes of gum, lozenges, patches) were made to 6 still-enrolled centers during TTTF post-implementation. Across centers, 193 clients and 13 employees received 495 and 54 boxes of NRT, respectively. Thus, the goal of 80% of centers reporting tobacco use assessments delivery and NRT dispensation to clients through 6 months post-implementation, relevant to only 6 of the centers, was achieved (6/6 centers = 100% for tobacco use assessments; 5/6 centers for NRT = 83.33%; Table 4).

## 4. Discussion

Tobacco use is exceedingly high among individuals receiving care for OUD, but rarely addressed by clinicians in these treatment settings [9,10,11]. Although prior studies implemented a similar program in addiction/substance use treatment centers [25,31,35,43,44,45,46], to our knowledge, no prior studies have reported details of the implementation of multi-component, multi-level treatment programs designed to build capacity for addressing tobacco use within OUD treatment centers. The current study addressed this gap by describing outcomes of the implementation of TTTF, a comprehensive tobacco-free workplace program [24,25,26,27,28,30,32], within 7 OUD treatment centers in Texas. Overall, 84.7% (6/7) of enrolled centers completed the program. Program goals were structured according to the RE-AIM framework. Specific subgoals included: educational reach to employees, increased exposure to tobacco education constructs, and clinician attendance at Certified Tobacco Treatment Specialist and Motivational Interviewing training (Reach, Goal 1); achieving employee knowledge gains from education and reduction in tobacco using employees (Effectiveness, Goal 2); completion of the TTTF program and implementation of a tobacco-free workplace policy with permanent signage (Adoption, Goal 3); changes in clinician behaviors in addressing tobacco use with clients (Implementation, Goal 4); and sustained provision of tobacco use assessments and NRT to clients over time (Maintenance, Goal 5). Of the RE-AIM framework program goals, all subgoals categorized under Adoption (Goal 3) and Maintenance (Goal 5) were achieved. Reach (Goal 1), Effectiveness (Goal 2), and Implementation (Goal 4) subgoals were only partially achieved.

Prior studies have shown that clients and employees alike perceived tobacco dependence treatment as a barrier to delivering effective treatment for other substance use disorders [47,48]. Our education program, which reached 64.56% of all employees, provided opposing data, significantly bolstering knowledge about the harms of tobacco use to individuals with OUDs and the importance of actively addressing nicotine addiction in chemical dependency treatment. Results indicated increased exposure to training about tobacco use, with significant increases seen in exposure to the hazards of smoking and benefits of quitting that are specific to individuals with substance use disorders and, in particular, OUDs. The provision of this education preceded the implementation of a tobacco-free workplace policy and was intended to provide background on how this, and other evidence-based methods, could meaningfully address the problem of tobacco use among OUD patients. The education program was also intended to increase employees’ knowledge about tobacco use and treatment, which was achieved with a significant, 54.9% percent increase in knowledge from pre- to post-education. These results are largely congruent with existing literature supporting that general education improves short-term knowledge, acceptability of, and attitudes towards tobacco-free workplace policies [29]. While highly promising, non-profit substance use treatment centers risk significant loss of knowledge with employee turnover, as they are often under-resourced and small in size [31,41]. As such, it is important for treatment centers implementing a program like TTTF to have a mechanism in place for continued employee training on tobacco use and dependence. One such model can be found on our website [49], which provides program champions with a curriculum and guidance to lead long-term training efforts. Given that clinicians frequently endorse time constraints and competing clinical priorities as an obstacle to attending trainings and to addressing tobacco use among clients [50], it is imperative that center leadership mandates and supports such efforts. Future studies should seek to determine the ideal timing and frequency of continuing education efforts to preserve knowledge gain within these settings.

In the current study, clinicians exhibited significant increases in all tobacco education constructs assessed (9/9). However, only 3 out of 7 centers were able to send a clinician to Certified Tobacco Treatment Specialist training and only 2 out of 7 took advantage of Motivational Interviewing training. Given provided resources were free, these were missed opportunities to embed specialized knowledge within the center that could serve as an ongoing resource to colleagues and clients. Moreover, although some centers cited prior Motivational Interviewing training as a reason to forgo attendance, studies show that expertise in its delivery is enhanced by continued training and coaching opportunities [51]. Center leadership endorsed intention to comply with these program requirements through the memorandums of understanding; however, compliance was not achieved. As an external entity attempting to change the organizational, clinical, and employee status quo regarding addressing tobacco use, it can be difficult to balance need to preserve a positive relationship with participating centers versus insisting upon adherence to an original agreement (cf. [52]), particularly during a pandemic. However, more work is needed on this front to ensure full center participation in important evidence-based aspects of a comprehensive tobacco control program, as trainings can impact clinician behavior change [28,53]. Likewise, the accuracy of reported knowledge gains across our implementation period may be limited by high turnover rates in these frequently underfunded and understaffed substance use treatment centers [35,36] where high employee turnover rates are common [54]. In addition, pre- to post-surveys measuring educational exposure allowed for clinicians and employees to skip questions that were not related to their job role; as a result, differences between missing data and skipped questions could not be distinguished. Future studies replicating this work should include a “Not Applicable” option to address this issue. Regardless, demonstrated increases in knowledge and training exposure are important, as translation of this information into actions with clientele is pivotal in impacting tobacco use rates.

Results of our study indicated self-reported increases among clinicians in delivery of the 5A’s whereby 74.1% to 96.2% of clinicians reporting engaging in their use post-intervention. While these gains relative to pre-intervention practices did not reach statistical significance, rates of Asking about tobacco use, Advising clients to quit, and Assisting clients to quit were marginally significant (*ps* < 0.10). As seen in other work [24,30], the lowest engagement was found for assisting with quit attempts and arranging follow-up, both endorsed by 74.1% of responding clinicians. These lower percentages may be related to encounters with clients who are not interested in quitting and thus would not need assistance or arrangement for follow-up [30]. Alternatively, clinician specific roles may be limited in scope for tobacco treatment; thus, referral may have served as a terminal intervention for smoking [30]. Regardless, these results suggest opportunities for additional capacity-building beyond assessment of interest in quitting. Notably, all still-enrolled centers started delivering tobacco use assessments during the program and all centers continued to do so post-program implementation. Identifying tobacco-use status via tobacco use assessments is consistent with best practices for tobacco control and guides clinicians to identify the most appropriate interventions for clients [39]. Given that many smokers endorse a clinician’s advice as a major motivator to quit, this point-of-contact is necessary for facilitating successful quit attempts [39].

Increases were seen over time in the provision of all specific cessation treatments; however, only the provision or referral/recommendation of NRT—along with a decrease in doing nothing to address tobacco use—met statistical significance. These results are consistent with TTTF’s provision of NRT to centers both during and post-implementation. Although we made recommendations to centers on this point, longer-term data collection is necessary to assess the extent to which centers built NRT expenses into their annual budgets or otherwise found sources of support for continued provision beyond our starter kits. Clinicians have frequently cited the value of NRT in facilitating client quit attempts during implementation [24,27]. Thus, having NRT conveniently available is necessary to facilitate quit attempts and ultimately change center smoking culture, as previous work has demonstrated that clients are more inclined to try freely available NRT [25,55].

Self-reported information from clinicians also reveals areas in need of additional work. Specifically, provision of “behavioral counseling” and “non-nicotine-based mediations or referral for such” were endorsed by only 39.3% and 14.3% of clinicians post-implementation. Ideally, clinicians should not rely on a single available method of treatment (NRT), but instead be comfortable with a range of possibilities—particularly in provision of behavioral counseling. While it is not clear why clinicians in these settings did not widely adopt behavioral counseling or non-nicotine-based medications in their practice, it is evident that there is room for improvement. Clinician compliance in these areas may be improved in future work with qualitative data to better understand barriers to the adoption and use of these interventions. Future studies may also seek to collect client-level data to help contextualize treatment and implementation-related outcomes and potentially improve uptake of certain evidence-based practices by clinicians. Although this was not part of the current work’s methodology, other studies have been able to collect individual-level data to support their implementation. For example, a study looking at New York’s statewide organizational tobacco control programs found significant decreases in smoking among clients in methadone-providing centers, following increases in the provision of NRT and other tobacco-related services [56]. While these data may enhance the ability to gauge the true effects of tobacco-free workplace policies and programs, increases in the 5A’s and other empirically supported interventions for tobacco may certainly have clinical significance and be indicative of a critical paradigm shift in treatment culture within these settings [57]. While changing clinician behaviors in addressing tobacco use is promising, the increased dissemination of evidence-based tobacco treatment is a critical area of improvement for TTTF implementation in OUD treatment settings.

Finally, although this was not an explicit program goal, all participating centers ordered passive dissemination materials encouraging quit attempts from TTTF for posting or distribution to their clientele. As prior work has suggested that clinicians view a paucity of passive dissemination materials as a barrier to screening and treatment of tobacco [50], the interest in and receipt of these materials was desirable. Additionally, given the often higher tobacco use rates among clinicians treating individuals with substance use disorders [57], provision and uptake of NRT by employees is a positive sign of engagement with the program, and could further change the pro-tobacco use culture at these centers.

A particular limitation of using the RE-AIM framework to evaluate TTTF’s implementation was the definition of the specific subgoals that maybe somewhat idiosyncratic to the present work. For example, a pragmatic application of RE-AIM for tobacco treatment programs was suggested for the field, but this came out following our funding and was not used to shape our methodology [58]. Future studies in this area, however, should strive for consistency with such models to facilitate comparability between studies.

Other limitations include an anonymous data collection procedure that prevented linking pre- and post-responses within employees, and non-response rates that may affect the generalization of results. Specifically, the survey response rate was low (50–60%) when considering the potential number of eligible employees in enrolled centers (although the item response rate was much higher at >80% when only the number of employees responding to a survey was taken into consideration). It may be that there was non-response bias wherein participants who responded differed from those who did not. In addition, the total number of employees of the enrolled centers was reported prior to program implementation and not assessed again during the multi-month roll-out, which might have yielded imprecise post-implementation survey response rates using the number of eligible staff from the pre-implementation data as the denominator. This is especially possible given the known high staff turnover in substance use treatment settings [54]; thus, those who participated in pre-implementation data collection might not have been employed at the time of post-implementation data collection, which might in part explain why our post-implementation response rates were so low [54]. In TTTF’s ongoing and future projects, we have improved our procedures to account for the number of employees at the time of the post-implementation survey release for enhanced accuracy.

This potential for non-response bias suggests that the findings should be interpreted with some caution and within the context of the limitations of this research outlined above [59,60]. For instance, it is possible that the employees who responded had the most interest in or commitment to enact changes related to tobacco treatment. It is vitally important to interpret conclusions drawn from Goals 1, 2, and 4 in light of potential non-response bias. For example, for Goal 1, it is possible that the exposure to training and education might have in fact been even lower if we had higher rates of responses. This is potentially true if missing staff/clinicians did have lower interest/commitment and thus would have been less likely to attend these sessions. In line with this, for Goal 2, it is possible that higher rates of participation would have yielded lower increases in tobacco knowledge if the missing employees were not engaged. However, it is also possible that higher rates of participation would have in fact led to increased knowledge gains, as less interested/committed employees may also have lower levels of baseline knowledge about tobacco use. Likewise, Goal 4 of clinician behavior change could be influenced either positively or negatively if the missing clinicians had different levels of interest/commitment. However, it seems most reasonable to assume clinicians with the most interest in the provision of tobacco treatment remained engaged in the program post-implementation. Therefore, it is possible that our high levels of 5A’s were reflective of dropout of uninterested clinicians (rather than an effect of our intervention). Unfortunately, these speculations cannot be empirically confirmed without collecting employee characteristic data. Future studies should aim to collect these data as part of evaluation procedures and consider examining more precisely the reasons behind non-participation, potentially using qualitative methods.

Finally, there is the possibility that for the part of Goal 2 relevant to decreases in employee tobacco use that social desirability biases (i.e., wanting to portray a positive picture of oneself) could have influenced responses to suggest more favorable use rates. We mitigated this possibility by not explicitly communicating to partnering centers the goals of the program regarding reducing employee tobacco use. However, implementing a deidentified or coded data collection method in future implementations may help to further establish the reliability and validity of self-reported measures from employees, such as tobacco use, that could then subsequently stimulate tailored follow-ups for improvement in various program areas (e.g., biochemical verification of smoking status).

## 5. Conclusions

In conclusion, TTTF’s implementation within OUD treatment settings affected both clinical and policy changes consistent with tobacco control best practices [61,62]. Overall findings are consistent with prior implementation in behavioral health centers [28], which are typically larger in scale and better resourced to tackle tobacco use. As such, these results demonstrate promise for an integrated approach to tobacco cessation treatment within OUD treatment settings and suggest that TTTF’s implementation may be a feasible model to guide future efforts within similar settings. However, study limitations, including lower post-implementation evaluation response rates, suggest that results require replication in other opioid addiction treatment settings.

## Figures and Tables

**Table 1:** Employees and annual client contacts in enrolled opioid addiction treatment centers in Texas, US.

Center	Clinicians	General Staff	Unique Clients Served	Annual Contacts
1	15	0	199	15,572
2	10	0	45	3521
3	6	2	170	13,300
4	7	3	100	7825
5	15	0	350	50,000
6	12	3	256	5869
7 *	6	0	75	20,000
**Total**	71	8	1195	116,087
**Total for completing centers**	65	8	1120	96,087

Note: * = center dropped out due to due to insufficient resources and competing priorities as the result of the COVID-19 pandemic following the provision of tobacco control and treatment training.

**Table 2:** Training receipt from pre- to post-implementation among responding employees and clinicians in opioid addiction treatment centers.

	Training Query Items	Pre	Post	*p*-Value
	In the Last 12 Months, Have You Received Any Training On…	% (*n*) Endorsing Yes *		
	***n***	32	22	
All Employees (5 Treatment Centers) ^§^	Any education regarding the hazards of smoking ^╪^ (Pre: 87.5%; Post: 81.8%)	64.3 (18)	72.2 (13)	0.5794
	The hazards of smoking and benefits of quitting that are specific to individuals with substance use disorders ^╪^ (Pre: 87.5%; Post: 81.8%)	67.9 (19)	83.3 (15)	0.2486
	***n***	36	28	
Clinicians-only	Assessing clients for their tobacco use ^╪^ (Pre: 86.1%; Post: 96.4%)	35.5 (11)	85.2 (23)	0.0002
(6 Treatment Centers)	Treating tobacco use in conjunction with SUDs ^╪^ (Pre: 86.1%; Post: 96.4%)	38.7 (12)	85.2 (23)	0.0005
	How quitting tobacco improves substance use recovery ^╪^ (Pre: 83.3%; Post: 92.9%)	43.3 (13)	84.6 (22)	0.001
	How continued substance use may be a barrier to successfully quitting tobacco ^╪^ (Pre: 86.1%; Post: 96.4%)	48.4 (15)	85.2 (23)	0.0045
	The use of pharmacotherapies (e.g., NRT, Chantix) to treat tobacco use ^╪^ (Pre: 86.1%; Post: 96.4%)	38.7 (12)	85.2 (23)	0.0005
	The effects of tobacco smoke on psychiatric medications ^╪^ (Pre: 86.1%; Post: 96.4%)	32.3 (10)	66.7 (18)	0.0118
	How tobacco may be used to cope with the side effects of psychiatric medications ^╪^ (Pre: 83.3%; Post: 96.4%)	20.0 (6)	59.3 (16)	0.0077
	The hazards of smoking and benefits of quitting specific to individuals with SUDs ^╪^ (Pre: 86.1%; Post: 96.4%)	48.4 (15)	85.2 (23)	0.0019
	The use of counseling and behavior therapies to treat tobacco use (e.g., MI) ^╪^ (Pre: 83.3%; Post: 92.9%)	43.3 (13)	84.6 (22)	0.0055

Note: * Respondents could skip items not relevant to their job duties; thus, percentages are calculated based on the number of item respondents. SUDs = Substance Use Disorders. NRT = Nicotine Replacement Therapies. MI = Motivational Interviewing; ^╪^ item response rate. ^§^ One center (Center 1) was excluded from the pre- and post-implementation comparison analyses, as this center did not provide post-implementation employee data, citing very high rates of employee turnover.

**Table 3:** Intervention provision from pre- to post-implementation among responding clinicians (6 opioid addiction treatment centers).

Intervention Query Items	Pre	Post	*p*-Value
With Regard to Clientele that You Saw Last Month Who Smoked, Did You…	% (*n*) Endorsing Yes *		
Ask clientele about their smoking status? ^╪^ (Pre: 91.7%; Post: 96.4%)	78.8 (26)	92.6 (25)	0.0758
Advise them to quit smoking? ^╪^ (Pre: 83.3%; Post: 92.9%)	83.3 (25)	96.2 (25)	0.094
Assess their willingness to make a quit attempt? ^╪^ (Pre: 83.3%; Post: 96.4%)	80.0 (24)	85.2 (23)	0.3491
Assist them to quit by providing treatment or making a referral for treatment? ^╪^ (Pre: 83.3%; Post: 96.4%)	53.3 (16)	74.1 (20)	0.0553
Arrange to follow up with them to assess their progress regarding smoking cessation? ^╪^ (Pre: 83.3%; Post: 96.4%)	63.3 (19)	74.1 (20)	0.2827
**What types of treatment do you typically provide for cigarette smokers and/or other tobacco users?**	% (*n*) Endorsing Yes *		***p*-value**
Behavioral counseling	25.0 (9)	39.3 (11)	0.2213
Nicotine replacement therapy (e.g., nicotine patch, gum) or referral/recommendation for such	22.2 (8)	71.4 (20)	<0.0001
Non-nicotine based medications (e.g., Chantix) or referral for such	8.3 (3)	14.3 (4)	0.6894
I do not typically provide treatment for smokers or other tobacco users	47.2 (17)	21.4 (6)	0.0329

Note: * Respondents could skip items not relevant to their job duties; thus, percentages are calculated based on the number of item respondents; NRT: nicotine replacement therapies; ^╪^ item response rate.

**Table 4:** Tobacco Use Assessments (TUAs) Delivered to Clients and Nicotine Replacement Therapy (NRT) Boxes Dispensed to Clients and Employees of Participating Opioid Treatment Centers Post-Program Implementation.

Center	Implementation		TUA Delivery	Boxes of NRT Dispensed			
	Start Date	End Date		To Clients	# Clients	To Employees	# Employees
1	06/2019	01/2020	58	0	0	0	0
2	09/2019	04/2020	50	293	120	23	5
3	09/2019	04/2020	46	28	20	3	2
4	10/2019	07/2020	5	15	15	0	0
5	10/2019	04/2020	33	139	33	28	6
6	05/2020	01/2021	80	20	5	0	0
7 *	02/2020	N/A	-	-	-	-	-
**Total**			272	495	193	54	13

Note: Start Date = date memorandum of understanding signed; End Date = final month in the 6-month post-implementation range; * = center dropped out due to insufficient resources and competing priorities related to the COVID-19 pandemic, following provision of tobacco control and treatment training.

## Data Availability

The data presented in this study are available on request from the corresponding author. The data are not publicly available due to confidentiality/privacy agreements with funders.
